# Bloodworm induced Anaphylaxis

**DOI:** 10.1186/1710-1492-10-S1-A29

**Published:** 2014-03-03

**Authors:** Peter Ho, Chrystyna Kalicinsky

**Affiliations:** 1 Section of Allergy and Clinical Immunology, Department of Internal Medicine, University of Manitoba, Winnipeg, Manitoba, Canada, R3A 1R9

## Introduction

Bloodworms are a group of bristle worms found at the bottom of marine waters. They have pale skin, which allows their red body fluid, which contains hemoglobin, to show through, hence the name “bloodworm”. Bloodworms are often used as bait in fishing and are also available commercially as a food-source for aquarium fish [1,2].

This case report describes a patient who experienced repeated allergic reactions after feeding her fish.

## Case report

A 21 year old female student was seen in the Adult Allergy and Immunology clinic for evaluation of hives. Eleven months ago, the patient experienced three distinct episodes where she experienced hives and itching affecting her neck. The first episode lasted for 30 minutes and then spontaneously resolved. Her second episode was similar to the first, but she also experienced the sensation of throat closing. She denied tongue or lip swelling and resolution occurred within 30 minutes. Her third episode was similar to her second episode and in addition she experienced itchy eyes. When the patient was asked about any suspected triggers, should reported that these symptoms occurred shortly after feeding her fish with bloodworms. After she suspected bloodworms as the etiology for her reactions, she switched fish food to mosquito larvae and has had no further recurrence.

The patient had epicutaneous skin testing to a solution of bloodworms in saline, which was positive.

## Discussion

This patient had recurrent urticaria as well as subjective respiratory compromise and conjunctivitis which occurred shortly after contact with bloodworms, with positive epicutaneous skin test. She meets the clinical diagnosis of anaphylaxis [3]. A literature search conducted in Pubmed with the keywords "Bloodworm Allergy" in July 2013 identified a case report of blood-worm induced asthma, but did not reveal any published reports of blood-worm induced anaphylaxis [4]. We counseled the patient to avoid any further contact with Bloodworms and to continue with an alternative fish food.
